# *On est ensemble*: social capital and maternal health care use in rural Cameroon

**DOI:** 10.1186/s12992-015-0121-0

**Published:** 2015-08-01

**Authors:** Sarah McTavish, Spencer Moore

**Affiliations:** School of Kinesiology and Health Studies, Queen’s University, Kingston, Canada; Department of Health Promotion, Education, and Behavior, Arnold School of Public Health, University of South Carolina, 915 Greene St.; Discovery I, Room 529, Columbia, SC 29208 USA

## Abstract

**Background:**

Every day approximately 1500 women worldwide die due to pregnancy or childbirth related complications. Maternal health care use is critical in reducing maternal mortality worldwide. Cameroon has one of the highest maternal mortality rates worldwide, but there is little knowledge about maternal health care use in Cameroon, particularly in more remote areas. The purpose of this study was to examine the importance of social networks and social capital in maternal health care use in the Far-North province of Cameroon.

**Methods:**

A sample of 110 Cameroonian women was recruited door-to-door in the urban town of Maroua and rural village of Moutourwa in the Far-North province in 2009. A maternal health questionnaire was administered to women between the ages of 18-45. The questionnaire assessed maternal health care history, social network, and social demographic characteristics. Social capital was measured in terms of the average educational level of women’s networks. Bivariate and multivariable poisson regression analysis was used to assess the number of maternal health care visits as a function of social network characteristics, education, ethnicity, age, and urban and rural residence.

**Results:**

Among the 110 participants, 13 percent reported not having visited a health care provider during the last pregnancy – 19 percent of the women sampled in Moutourwa and 6 percent in Maroua. Findings showed that women with higher social capital had a greater tendency to use maternal health care services (IRR: 1.13; 95 % CI: 1.02-1.26). Social network size and social participation were not significant in full models. Ethnic characteristics were also shown associated with MHCU in the Far-North province.

**Conclusion:**

Although the size of women’s health-related networks were not shown significant, the resources to which women might have access through their social networks were associated with women’s maternal health care use in remote areas of Cameroon. Although pregnancy may not be widely discussed in public, women’s social networks may provide key social resources, e.g., information or financial capital, that facilitate MHCU. Leveraging women’s social capital may provide a means to improve maternal health care use among women in low-income countries.

**On est ensemble:**

Social capital and participation in the use of maternal health care services in remote areas of Cameroon

## Introduction

Globally, there was an estimated 287,000 maternal deaths in 2010 with 99 % of those occurring in developing countries [1]. Sub- Saharan Africa had the highest maternal mortality ratio (MMR) in 2008 with 640 deaths per 100,000 live births [1, 2]. Major causes of maternal death include intrapartum emergencies such as haemorrhage, obstructed labour and infections. Approximately three-quarters of all maternal deaths are preventable [3]. Promoting the use of quality maternal health care services is considered a key strategy in decreasing maternal and infant mortality, especially in areas where the overall health status of women is low. Research has shown that women with higher levels of education are much more likely to use health care services [4]. Despite the recognized importance of women’s own personal education for maternal health care use (MHCU), less is known about the importance of women’s social networks and the role that social capital may play in women’s access to maternal health care (MHC).

The objectives of this study were to assess the importance of women’s social networks, social capital, and social participation in accessing MHC services, and whether women with higher social capital and participation were more likely to use MHC services. Understanding the role that social and interpersonal factors play in MHCU can aid in the design of maternal health promotion programs that target the clustering of non-MHCU among women. Social networks refer to the pattern of social ties existing among a set of actors; actors may be individuals, organizations, or other types of entities; social ties may consist of various types of relationships, including friendship, kinship, or the exchange of specific types of information or other resources. Networks can enable the sharing of information and advice about care during pregnancy and childbirth [5, 6], and have been suggested to be the principal mechanism through which health-related decision occurs [7]. Social capital refers to the resources that individuals or groups are able to access through their social networks [8]. Research on social capital and health has often defined social capital along three different dimensions: (1) network resources to which individuals or groups have access; (2) the cognitive, psychosocial resources (e.g., generalized trust, reciprocity, perceived social cohesion) that characterize geographical units; and, (3) the structural opportunities that facilitate social capital formation (e.g., social participation) [8]. While different disciplines and scholars (e.g, Putnam, Coleman, and Bourdieu) often highlight various dimensions of the social capital concept, much research has aimed to differentiate these dimensions to assess whether one may be more important than another for a particular outcome.

This study assumes a network approach to social capital. Whereas social networks structure the exchange of physical, emotional, informational, and material resources, the construct of network social capital may be seen to capture the quality and diversity of socioeconomic resources that may be accessed through social connections [9, 10]. These resources may be influential in the choices that women make regarding their health. For example, Gage [5] has suggested that social networks characterized by high levels of education facilitate women’s access to information about health services and minimize their uncertainty about medical systems. The educational level characterizing women’s interpersonal networks may thus reflect their accessibility to key social resources. Green and Rodgers [11] have also suggested that network members can offer support emotional or instrumental support through health advice, providing childcare, giving parenting guidance or showing sympathy and offering encouragement [11]. As a structural dimension of social capital, social participation captures a person’s level of engagement in formal and informal social and civic associations or activities. Research has shown that individuals with higher levels of engagement tend to have more health promoting behaviors [12]. Community-level participation has also been shown to have positive impacts on maternal and child health [13]. Nevertheless, despite research mainly showing the benefits of social participation for health, social capital and participation can also have negative consequences [14, 15]. For example, research from South Africa has suggested that social engagement in disadvantaged settings without sufficient resources or capacity can have negative repercussions for health. In such contexts, cultural norms may dissuade women from acting autonomously to address mental and HIV-related health problems [16].

Social networks, social capital, social participation may be particularly relevant factors for women accessing MHC in rural, developing country settings where medical and health resources are scarce, relatively expensive, and often distant from places of residence. In such contexts, social capital may lead to disparities in MHCU in that women with greater social capital may be able to leverage their social resources to access more scarce and distant services. The Far-North province of Cameroon is located approximately 500 km from the administrative capital of Yaoundé and the financial capital of Douala. The province lacks the maternal health care resources of other areas of Cameroon. Medical professionals are often unwilling to work in the north due to its isolation, lack of resources and extreme temperatures. The Far-North province is ethnically and culturally heterogeneous with polygamy as a common practice leading to multiple wives in a household [17, 18]. Regardless of women’s marital status or arrangement, women tend to rely on other female sources of support for information and help with home remedies, family care, and support and treatment of certain reproductive health problems [18]. Research has suggested that the spatial clustering in women’s use of prenatal care may reflect the social network clustering of women’s use of care [5].

### Cultural context of social network influences

Social networks are embedded in specific cultural and historical contexts. These contexts can shape the composition and operation of social networks and the types of resources exchanged and circulated within networks [19]. In the Far-North province, there is a hesitation to speak openly about pregnancy, especially in the first trimester, before the pregnancy becomes visually obvious [17]. There is often fear that envious women can cause harm to pregnant women through witchcraft. Expectant mothers will often conceal their condition as long as possible to avoid the negative outcomes of witchcraft, such as miscarriage and stillbirth [17]. Because of the hesitation to speak about pregnancy, women tend to be selective about whom they talk to and tend to discuss their health during pregnancy with very few people. Rather than sharing news about the pregnancy or seeking information from a range of individuals, women may restrict their information seeking to only a limited few within their close networks. As such, network size may be of lesser importance for women’s MHCU than the quality of social capital available to women through their networks. Furthermore, studies have shown that household education, including the husband’s educational level, significantly increases women’s use of prenatal services and delivery in a health care facility [20, 21]. As such, having higher social capital may be more important than a woman’s own level of education in whether they use prenatal and maternal health care services.

## Methods

### Setting and sample

This study is based on a convenience sample of 110 women between 18-45 years old who had given birth at any time in the five years prior to September 2009 (*M* age = 28.5 years, *SD* = 6.3). The lead author carried out two months of intensive fieldwork in the Far-North province of Cameroon during July and August 2009. Two fieldwork sites were used for this study: Maroua, the provincial capital of the Far-North and Moutourwa, a rural community approximately 50 kilometres south of Maroua. The study aimed to recruit 60 participants from Maroua and Moutourwa. Only one woman per household was eligible. Maroua and Moutourwa were selected due to available logistic and research support and accessibility during the time of fieldwork (rainy season). Maroua has an estimated population of 200,000. It is the only city of its size in the province and the closest urban area to the Chadian capital, N’djamena. Moutourwa centre has a population of approximately 3000 inhabitants, and encompasses smaller villages like Ganaha and Badjava.

### Questionnaires

Questionnaires were administered to participants to elicit detailed information on their socio-demographic background, maternal health care practices and social networks. Local research assistants provided support in questionnaire administration. Participants might choose to receive information about the study and answer questions from the questionnaire in French, Fulfulde or Guiziga. Interviews were conducted wherever the participant felt most comfortable, usually in the home or within the family compound. Ethics approval was granted from Queen’s General Research Ethics Board (GREB) prior to research.

The maternal health care questionnaire had 35 items and contained three main sections: (1) personal history of maternal health care practices, satisfaction and perceived importance of using these services, (2) social support networks relevant to maternal health care, and (3) socio-demographic background. The questionnaire assessed number of live births, information about the most recent child born in the past five years, the location and type of care given during pregnancy and delivery, and the perceived importance that a mother placed on maternal health care. The section on social support networks consisted of three name generator questions and eight follow-up name interpreter questions. Name generator questions asked participants: 1) to name those with whom they discussed health and pregnancy information, 2) to name who they spoke to about job information or work, and 3) to name who would watch the household and or children if they had to leave the home. The name generator questions were designed to elicit the closest ties in each person’s network [22]. Name generator questions were followed by name interpreter questions that assessed compositional aspects of women’s networks, including relationship type (e.g., family/kin or friend), age, level of education, and information reciprocity within the network. Demographic questions asked participants about their level of formal educational attainment and age. Levels of education were categorized as 1) no formal education, 2) some primary education, 3) primary school completed, 4) some secondary school, 5) secondary school complete and 6) college or university attended. Participants were asked to provide their age or age range if they did not know their specific age.

To complement the structured questionnaire on maternal health service use, intensive semi-structured interviews lasting on average 35 min were undertaken with five participants in Moutourwa and four in Maroua. Follow-up interviews were limited due to overall research time constraints. Like the questionnaires, interviews took place at the participants’ homes, and were conducted in their preferred language with the help of local research assistants. Women were asked to discuss more broadly five aspects of their pregnancy: 1) food consumed during pregnancy; 2) who knew about the pregnancy and when, 3) any activities encouraged or discouraged during pregnancy 4) how pregnant women were viewed in the community and 5) their experience of delivering at home or in the clinic. Interviews conducted in Fulfulde or Guiziga were first orally translated into French by one of the research assistants; interviews conducted originally in French or later translated into French were translated into English by the lead author.

### Measures

#### Maternal Health Care Use

The outcome variable of number of maternal health care visits was the number of times a woman reported having seen a health care provider during her last pregnancy. Distinctions were not made on the trimester of pregnancy in which the visit(s) were made or the specific type of care provided.

Social capital and network characteristics: Name generator/interpreter information on those alters with whom participants specifically discussed their health and pregnancy was used for this study. Network size was based on the number of persons that they nominated as part of their health and pregnancy network, with a possible range from 0-5. Social capital was measured in terms of the alters’ average level of educational attainment. Unlike human capital, which would be more appropriately represented as the participant’s educational level, our measure of social capital is meant to signify the quality of socioeconomic resources that a participant might have access through their social networks. Social participation was based on whether female study participants reported being involved in any women’s groups, such as mother’s associations or religious groups.

Demographic and confounding variables: Among participants, 34 % reported not knowing their year of birth. However, all women reported the age group in which they considered themselves belonging. Age was thus defined categorically based on the age range that women reported: (1) 18-19, (2) 20-29, (3) 30-39, and (4) 40-49 years. Analyses showed that age did not have a curvilinear relationship with MHCU and so was kept in categorical form. Urban and rural residence was based on whether the participants lived in Maroua or Moutourwa respectively. Given participants’ low levels of education and its skewed distribution, participants’ educational level was dichotomized into no education and some level of education for these analyses. Women reported the number of previous births to which they had given. Ethnicity was also self-reported, and grouped into seven categories: (1) Fulbe, (2) Kirdi, (3) Mundang, (4) Tupuri, (5) Guiziga, (6) Guidar, and (7) Other. Analyses showed that the Mundang had higher frequency of MHCU than other groups, so analyses contrasted the MHCU of the Mundang against all other ethnic groups. Whether women were part of a polygamous marriage or not was also assessed in bivariate models but not included or reported here due to its non-significance.

### Analyses

Poisson regression was used to examine the association among the number of maternal health care visits, social network and capital characteristics, age, personal education level, urban/rural residence, number of previous births, and self-reported ethnicity. Three sets of regression analyses were conducted. First, the bivariate associations between MHCU and study variables were assessed (Model 1). Second, the association between MHCU and social capital characteristics were assessed while adjusting for social network size (Model 2). Finally, the association between MHCU and women’s social capital was assessed, while adjusting for possible confounding variables. Incidence rate ratios (IRRs) were computed and reported along with 95 % confidence intervals (CIs). Regression analyses were conducted using Stata version 14.

Using NVivo software, inductive content analysis was conducted on all interview transcripts to discover patterns, themes and categories in the data. Content analysis included both pattern and thematic analysis. Reoccurring descriptive findings or patterns were used to identify themes [23], with open coding used to identify actions, feelings and events in the data. For the purposes of this study, we use qualitative findings to complement the interpretation of the empirical results in the discussion section. For additional information about the qualitative content analysis and results from these interviews, please see McTavish [24].

## Results

Table 1 provides descriptive information on the demographic, social network and maternal health care use characteristics of the sample. Participants came from six different neighbourhoods in Maroua (n = 50) and five in Moutourwa (n = 60). Among the 110 participants, 13 percent reported not seeing a health care provider during their last pregnancy – 19 percent of the women sampled in Moutourwa and only 6 percent in Maroua. Women in Moutourwa reported on average 4.8 previous births, whereas women in Maroua reported 3.2. Of the women sampled, 37 percent had no formal education in Moutourwa, whereas in Maroua only 10 percent of women had never been to school. In Moutourwa, 53 percent of the women sampled gave birth at the local hospital and 47 percent gave birth either at their own home or at their parent’s home. The Tupuri and Mundang had the greatest percentage of ethnic representation in the Maroua sample, and the Guizaga in the Moutourwa. Of the 110 participants, 10 percent reported not speaking to anyone about their health or pregnancy during their last pregnancy – 13 percent in Moutourwa and 6 percent in Maroua. According to their name generator data, women in Maroua spoke on average to 2.16 people about their pregnancy, whereas women in Moutourwa spoke to 2.03 people. Of those named, roughly 37 percent in the Moutourwa sample had no formal education compared to 6 percent in the Maroua sample. The majority of women in both locations reported their husbands as among those with whom they had spoken with about their pregnancy. In Maroua, only 44 percent of women belonged to a women’s group, whereas 53 percent did in Moutourwa.

**Table 1:** Descriptive statistics of maternal health care use and social networks in Maroua and Moutourwa, Cameroon, n = 110

	Maroua	Moutourwa
	n = 50	n = 60
MHC visits (Mean/SD)	5.5 (3.0)	3.6 (2.1)
Social network size (Mean/SD)	2.2 (1.4)	2.0 (1.4)
Network education		
No formal education	6.2 %	37.2 %
Some primary	28.5 %	33.5 %
Primary completed	2.0 %	0
Some secondary	43.8 %	18.7 %
Secondary completed and more	19.4 %	10.6 %
Active Women’s group member	44.2 %	53.6 %
Age range		
18-19	3.8 %	9.7 %
20-29	62.3 %	38.2 %
30-39	29.7 %	42.5 %
40-49	4.2 %	9.6 %
Education		
No formal education	9.7 %	36.5 %
Some primary	39.9 %	41.6 %
Primary completed	0 %	0.3 %
Some secondary	40.3 %	21.4 %
Secondary completed and more	8.1 %	0.2 %
Previous number of births	3.2 (2.4)	4.8 (2.9)
Ethnicity		
Fulbe	6.0 %	5.0 %
Mundang	18.0 %	5.0 %
Tupuri	20.0 %	1.7 %
Guizaga	12.0 %	83.3 %
Guidar	4.0 %	1.7 %
Other groups	40.0 %	3.3 %

**Table 2:** Incidence rate ratios from bivariable and multivariable poisson regression of the number of maternal health care visits on study variables in Maroua and Moutourwa, Cameroon, n = 110

Variables	Bivariate	Model 1	Model 2
	IRR	IRR	IRR
	(95 % CI)	(95 % CI)	(95 % CI)
Network size	0.98 (0.94-1.03)	1.00 (0.95-1.03)	1.00 (0.95-1.04)
Network resources (Alter education)	1.28*** (1.18-1.38)	1.27*** (1.17-1.38)	1.13** (1.02-1.26)
Social participation			
Active	1.26** (1.06-1.51)	1.19 (1.00-1.43)	1.20 (0.97-1.49)
Non-active	1.00	1.00	1.00
Setting			
Rural	0.67*** (0.56-0.80)		0.83 (0.67-1.03)
Urban	1.00		1.00
Education			
No education	0.61*** (0.48-0.78)		0.90 (0.67-1.21)
Some education	1.00		1.00
Age Category	0.94 (0.83-1.06)		0.92 (0.77-1.11)
Number of previous births	0.96* (0.93-0.99)		1.00 (0.96-1.05)
Ethnicity			
Mundang ethnicity	2.00*** (1.60-2.50)		1.51** (1.16-1.96)
All other ethnicities	1.00		

***p < 0.001, **p < 0.01, *p < 0.05

Table 2 provides the results of the bivariate and multivariate poisson regression models. In the bivariate models, network size was not associated with the number of times women saw a health care provider. Social capital (IRR: 1.28; 95 % CI: 1.18-1.38) and social participation (IRR: 1.26; 95 % CI: 1.06-1.51) were each positively associated with MHCU in bivariate analyses, meaning that women with a higher network socioeconomic resources and greater participation also tended to more frequently use maternal health care services during their last pregnancy. Women in rural Moutourwa (IRR: 0.67; 95 % CI: 0.56-0.80), those with no education (IRR: 0.61; 95 % CI: 0.48-0.78), or have had a greater number of previous births (IRR: 0.96; 95 % CI: 0.93-0.99) were less likely to use maternal health services. Being Mundang (IRR: 2.00; 95 % CI: 1.60-2.50) was associated with greater MHCU. Model 2, adjusting for the social network and capital characteristics, showed only social capital associated with MHCU. In the fully adjusted model 3, MHCU was associated with only social capital (IRR: 1.13; 95 % CI: 1.02-1.26) and being Mundang (IRR: 1.51; 95 % CI: 1.16-1.96). Urban/rural setting and maternal education level were not significant.

## Discussion

The main objective of this study was to assess the importance of social network and social capital characteristics for women’s MHCU in the Far-North Cameroon. Numerous challenges and barriers exist in collecting social network data in rural, developing country settings, and little research has examined the importance of social networks and social capital for MHCU among rural women. Our research assessed the possible importance of social network size, social capital, and social participation in MHCU. Our findings showed that social network size itself was not associated with MHCU, but that the resources to which women might have access to through their social networks (i.e., social capital) were associated with greater MHCU. Social participation was associated with MHCU in bivariate but not multivariable models. Furthermore, maternal educational attainment was not significant in the final models, reflecting the possible importance of social over human capital in women’s use of maternal health care.

First, social networks have been shown in prior research to provide women access to information about maternal health care services and contacts to formal health care centres [5]. Our findings did not show an association between network size and MHCU. In the Far-North province, where polygamy is commonly practiced and families and extended families live together in compounds, information can circulate quickly due to the spatial proximity of living situations. Women with larger networks with whom they might share information regarding pregnancy and maternal health might thus be better able to access MHC services. For example, Magadi et al. [25] found that family social support networks tend to be greater in rural areas compared to large urban centres due to larger family support for childcare and to obtain maternal health care services. As such, social network size could potentially play an important role in women’s access to information and resources regarding maternal health care.

Local cultural practices and rules surrounding discussions of pregnancy may help explain why social network size itself was not important for MHCU. Qualitative interviews suggested that women who thought that they might be pregnant often refrained from discussing their condition. A false claim could cause embarrassment and talk in the community. Talking about one’s pregnancy could also result, according to certain participants, in unwanted attention that in some cases might cause harm to mother and child:

“You can’t tell just anyone that you are pregnant. There is a worry that if you tell too many people the baby will be born prematurely. The whole village will be saying ‘you are pregnant, when are you delivering?’ It is tiring.” [26].

There were many stories of miscarriages, still births and children being born with crippling illnesses because the mother was bewitched. Accusations of witchcraft can take place between strangers or women from the same family [27]. Disagreement between families can lead to resentment and acts of vengeance in the form of witchcraft. To protect themselves, some women reported telling only their husbands if they suspected that they were pregnant:

“There is no point in telling people you are pregnant – in time the body will speak for itself” [26].

Nevertheless, some women reported not even discussing matters with their husband if they felt that he did not have money to pay for the clinic or hospital visits.

Second, our research showed women’s social capital associated with MHCU. Women who were embedded in health-discussant networks with greater socioeconomic resources (as reflected through higher average educational attainment) had higher levels of MHCU. Research on social capital and health outcomes has highlighted a range of mechanisms by which social capital can improve health, including greater diversity in and access to information and material resources, social support, and improved social learning [28]. In this sense, a network of women who potentially understand the value of and possess resources to access maternal health care services, may be more likely to assist one another with childcare or provide financial assistance for transportation or medical costs [29]. Interestingly, women’s social capital was more strongly associated with MHCU than their own educational level. Prior research has shown that women’s education is associated with the increased use of prenatal care [20]. While women’s educational level is important for a range of women’s health outcomes, including MHCU in our bivariate analyses, our study highlights the importance of the interpersonal network context in which women are embedded.

Third, social participation was associated with MHCU in bivariate analyses but not when social network size and social capital were also included in the models. Little research has examined women’s social participation and their use of prenatal services, specifically in an African context [30]. Gayen and Raeside [30] suggested that women who belong to a social group such as a church group, community activist group or mother-teacher association are more likely to share or be influenced by group norms surrounding health care. Previous research has shown that women in Cameroon tend to belong to groups or networks with others like themselves [31]. It is likely that if prenatal services are used by the dominant members of the women’s groups, then other members will likely do the same. It was observed during fieldwork that in areas like Moutourwa where international NGOs and organizations are present, an increasing number of women were joining community women’s groups and mother-teacher associations. Community groups provide a forum for women to share information, socialize and potentially gain confidence to ask questions about their health, education and social rights. Women who socially participate may thus develop more resourceful networks that can be later leveraged for maternal health purposes. The reduced association between participation and MHCU may relate then to the possible mediating role that network resources play in the link between participation and MHCU. Future research with longitudinal data and a larger sample size would need to examine this issue in greater detail.

Besides identifying the possible role that social network and social capital may play in MHCU, our findings showed that the Mundang were more likely to access maternal health services than other ethnic groups in Cameroon. Many of the Mundang residing in Maroua were migrants from the village Lara, approximately 65 km away. Further research is required however to understand whether there may be specific cultural beliefs or practices that heighten the Mundang’s MHCU or whether there may be other factors at play, such as selective migration that could explain their greater use of maternal health services.

Three limitations are worth noting about the present study. First, the study is based on a convenience sample of women in Maroua and Moutourwa. Results may not be generalizable to other populations in Cameroon or other countries. Second, all data such as demographics, number of maternal health care visits, social network characteristics and women’s group participation were self-reported by participants. Since women were being asked about their last pregnancy, they may have misreported their use of maternal health care services. Third, the data are cross-sectional, which means for our analyses that we were unable to assess whether social capital causally influenced MHCU or MHCU might impact the types of networks that women might form.

## Conclusion

Despite its limitations, the study represents a unique examination of women’s social networks in rural areas of Africa. Findings may be suggestive and informative for future research and programs seeking to improve maternal health care use in developing countries. Promoting maternal health care use in resource-poor settings requires multidimensional and multilevel strategies that target the social ecological factors influencing women’s decisions about and access to maternal health care. In a cultural context in which the flow of information about pregnancy may be restricted and low levels of education exist, the resources to which women may have access through their social networks may be an important factor influencing women’s decisions about MHCU. Developing interventions that help to enrich women’s social capital resources could contribute to greater MHCU and improve other outcomes as well.
